# Interaction between Vitamin D homeostasis, gut microbiota, and central precocious puberty

**DOI:** 10.3389/fendo.2024.1449033

**Published:** 2024-12-09

**Authors:** Doudou Guo, Xin Ning, Tao Bai, Lingfang Tan, Yanfen Zhou, Zhichen Guo, Xin Li

**Affiliations:** ^1^ Department of Pediatrics, Union Hospital, Tongji Medical College, Huazhong University of Science and Technology, Wuhan, China; ^2^ Division of Gastroenterology, Union Hospital, Tongji Medical College, Huazhong University of Science and Technology, Wuhan, China

**Keywords:** central precocious puberty, vitamin D deficiency, gut microbiota, prevention, treatment

## Abstract

Central precocious puberty (CPP) is an endocrine disease in children, characterized by rapid genital development and secondary sexual characteristics before the age of eight in girls and nine in boys. The premature activation of the hypothalamic-pituitary-gonadal axis (HPGA) limits the height of patients in adulthood and is associated with a higher risk of breast cancer. How to prevent and improve the prognosis of CPP is an important problem. Vitamin D receptor (VDR) is widely expressed in the reproductive system, participates in the synthesis and function of regulatory sex hormones, and affects the development and function of gonads. In addition, gut microbiota plays an important role in human health by mainly regulating metabolites, energy homeostasis, and hormone regulation. This review aims to clarify the effect of vitamin D deficiency on the occurrence and development of CPP and explore the role of gut microbiota in it. Although evidence on the interaction between vitamin D deficiency, gut microbiota, and sexual development remains limited, vitamin D supplementation and gut microbiota interventions offer a promising, non-invasive strategy for managing CPP.

## Background

Precocious puberty (PP) is characterized by the onset of secondary sexual characteristics before age eight in girls and nine in boys, with central precocious puberty (CPP) being the most common form. CPP results from early activation of the hypothalamic-pituitary-gonadal axis (HPGA), and it affects girls 15-20 times more often than boys (1). This condition can impact adult height and is associated with an increased risk of type 2 diabetes, cardiovascular disease, breast cancer, and other complications in adulthood (2). While some causes of CPP are known—including genetic predispositions like KISS1 and MKRN3 gene variations, as well as endocrine-disrupting chemicals—many cases, particularly in girls (about 90%) and boys (up to 60%), remain idiopathic (3). There is also a global trend towards earlier puberty onset, necessitating better diagnostic and treatment approaches (4–7). While GnRH analogs remain the cornerstone of CPP treatment, novel sustained-release formulations and personalized therapeutic approaches are actively being developed.

Vitamin D plays a crucial role in children’s growth and development, maintaining calcium homeostasis, promoting bone growth, and regulating neuroendocrine and reproductive functions. The vitamin D receptor (VDR) is present throughout the hypothalamic-pituitary-gonadal axis (8–10). Adequate vitamin D levels are essential for normal pubertal progression and reproductive health, as demonstrated in mice (11). It significantly influences sex hormone secretion, gonadal development, and reproductive organ function. In humans, studies have linked vitamin D levels to the timing of menarche and overall reproductive health (12–15). Vitamin D deficiency (serum 25-hydroxyvitamin D below 50 nmol/L) is associated with various health issues, including immune dysfunction, obesity, metabolic syndrome, infections, cancer, and cardiovascular diseases (16). Recent meta-analyses indicate an inverse relationship between vitamin D levels and precocious puberty. However, findings on vitamin D deficiency prevalence in PP patients are inconsistent, possibly due to a threshold effect of vitamin D status (17–19).

The gut microbiota refers to the diverse community of microorganisms, including bacteria, archaea, viruses, and fungi, that inhabit the gastrointestinal tract. It plays a crucial role in metabolic, physiological, and immune functions and stabilizes to an adult-like composition within 1-3 years after birth (20, 21). Significant differences exist between the microbiota of children and adults, with microbial community structure, diversity, and functional potential varying by age and gender, especially during puberty (22, 23). The gut microbiota began to show gender differences during puberty and interacted with sex hormones, indicating that there was a relationship between gut microbiota and sexual maturity (24, 25). Additionally, vitamin D is vital for gastrointestinal health, influencing mucosal barriers, ILC3, and T cells, thereby affecting the microbiota (26). Both clinical and animal studies show that vitamin D can modulate the immune system through changes in gut microbiota composition and antimicrobial peptides (AMPs) regulation (27, 28). By promoting short-chain fatty acid (SCFA)-producing bacteria, vitamin D helps reduce inflammation, suggesting that vitamin D supplementation could restore gut homeostasis and offer therapeutic benefits for CPP (26).

Given the diagnostic and therapeutic challenges of CPP, there is an urgent need to explore its etiology further to possibly develop also non-invasive approaches. Recent studies highlight a potential link between PP, vitamin D, gut microbiota, and their metabolites, though the mechanisms remain unclear. This review aims to summarize the relationships between vitamin D, CPP, and gut microbiota, providing insights for future interventions through vitamin D supplementation and microbiota modulation.

## Methods

A systematic search of the PubMed database was conducted up to May 2024 to evaluate the literature on the interplay between CPP, vitamin D, and gut microbiota. The review includes original articles, meta-analyses, animal studies, and clinical studies. Search terms used were “central precocious puberty”, “pubertal disorders”, “age at menarche”, “timing of puberty”, “early menarche”, “puberty time”, “vitamin D”, “vitamin D3”, “vitamin D deficiency”, “microbiota”, “gut microbiota”, “microbiome”, “dysbiosis”, “sex hormones”, “short chain fatty acid”, and “bile acids”.

## The role and effect of vitamin D in central precocious puberty

Vitamin D’s role in CPP has garnered significant attention. It interacts with transcription factors, regulating vitamin D-sensitive genes critical for bone and mineral metabolism and other biological functions (29, 30). The vitamin D receptor (VDR) is widely distributed in somatic cells and organs, including bone, parathyroid glands, immune system components, and endocrine structures like the pancreas, hypothalamus, pituitary gland, and adrenal cortex. It is also present in reproductive tissues such as the testes, ovaries, and uterus (31, 32). Studies indicate that vitamin D is essential for neuroendocrine regulation, reproductive development, and immune function (33).

Studies in mammals have demonstrated that the active form of vitamin D, 1,25(OH)2D3, also known as calcitriol, stimulates the production of estradiol and estrone, while knockdown of the VDR significantly reduces testosterone synthesis and secretion in Leydig cells. This regulation may involve the expression of 3β-hydroxysteroid dehydrogenase (3β-HSD) and StAR (34–36). Vitamin D also promotes mitochondrial homeostasis, reduces oxidative stress and tissue damage, and regulates cellular health. Vitamin D deficiency, conversely, decreases mitochondrial activity and increases oxidative stress and inflammation (37). 1,25(OH)2D3 has immunomodulatory properties on T cells and can reduce pro-inflammatory cytokines such as IL-17, INF-γ, and TNF-α, exerting anti-inflammatory effects (38). Parathyroid hormone (PTH) indirectly affects vitamin D synthesis by regulating calcium levels, while vitamin D influences the secretion of pituitary gonadotropin through interactions with PTH and its receptors. Genetic factors are believed to account for 50-80% of the variation in puberty timing in the general population (39). Genome-wide association studies have identified numerous genetic loci influencing puberty timing across various ethnic groups, including loci associated with PP (40). Single nucleotide polymorphisms (SNP) are small modifications in the nucleotide sequence between individuals, and some SNPs may make subjects more susceptible to certain diseases. Recent genetic studies suggest a potential etiological role for SNPs in/near a custom list of genes related to nuclear hormone receptors including VDR during puberty (41). A case-control study by Li et al. indicated that VDR polymorphism may protect against CPP in Chinese girls by affecting the peak FSH level in the gonadotropin-releasing hormone (GnRH) stimulation test (42).

Preclinical studies show VDR expression in the hypothalamus, suggesting vitamin D may regulate reproductive system development by affecting GnRH neuron function. Vitamin D is involved in NMDA-mediated inhibition of GnRH neuronal activity, potentially delaying the onset of puberty (3, 43). Although vitamin D’s neuroprotective effects against reactive oxygen species (ROS) and inflammation are known, its specific mechanisms on HPGA remain unclear (44). Notably, vitamin D metabolic enzymes and VDR are expressed in white adipose tissue, and low-dose 1,25(OH)2-D3 inhibits apoptosis of differentiated 3T3-L1 adipocytes by regulating the expression of uncoupling protein 2 (45). Another study reports that 1,25(OH)2D3 mainly regulates the late stages of adipogenesis (46). This reveals a potential mechanism between obesity and CPP. Studies in female mice show that peripubertal vitamin D deficiency delays vaginal opening and estrus, while deficiency before weaning does not affect puberty (11, 47). VDR knockout mice exhibit gonadal dysfunction, including reduced sperm count and motility, and abnormalities in reproductive organs (48). Interaction among ANXA1, ANXA5, and VDR may influence gonadotropin secretion regulation in female rats (49).

**Figure 1:**
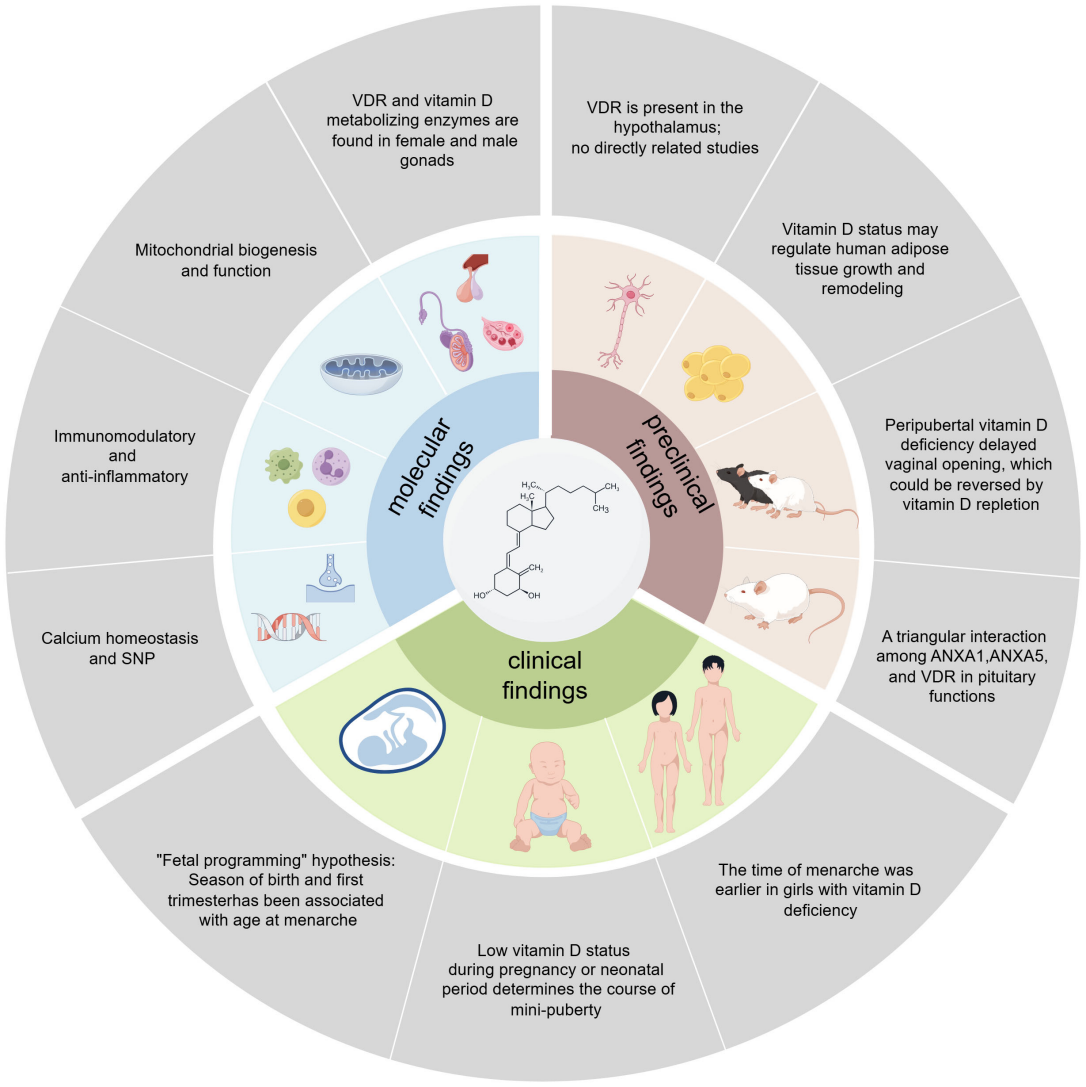
Summary of molecular, preclinical, and clinical findings linking vitamin D to puberty.

Consistent with the “fetal programming” hypothesis, several large-sample clinical birth cohort studies have found that the season of birth and first trimester with seasonal variations in endogenous vitamin D3 synthesis has been associated with age at menarche (50, 51). In addition, a single-center, matched cohort study found a low vitamin D status during pregnancy determines the course of mini-puberty in boys (52). Other evidence also suggests that 25(OH)D modestly affects total testosterone and inhibin B levels in girls during mini-puberty, indicating that 25(OH)D may influence gonadal function in early life (53). A case-control study found that vitamin D deficiency was associated with early menarche and was an independent risk factor for idiopathic central precocious puberty (ICPP) in girls (12, 54). Female gender and puberty were negatively associated with 25(OH)D (55). However, randomized clinical trials have yielded different results from animal studies, showing no significant effect of vitamin D treatment on testosterone or other hormone levels (56–58). This discrepancy may arise from not accounting for factors such as vitamin D-binding proteins and detection methods. Vitamin D deficiency might indirectly affect hormone status by regulating the bioavailable portion of testosterone, which requires further investigation considering age and the degree of vitamin D deficiency. In summary, the exact role and effect of vitamin D in the pathogenesis of CPP need further study to determine its specific mechanisms. The possible molecular mechanisms connecting vitamin D to puberty and the findings of preclinical and clinical studies are summarized in **Figure 1**.

## Difference in vitamin D concentration between the central precocious puberty group and the control group

It is postulated that a complex interplay of genetic, nutritional, and environmental factors may precipitate endocrine and physiological changes leading to puberty (3). Apart from potential adjustments in overall dietary patterns and lifestyle, certain nutrients, including vitamin D, may hold promise in influencing sexual maturation. However, there is no definitive consensus regarding the correlation between vitamin D and the onset and progression of CPP. Population-based epidemiological and clinical investigations suggest that serum vitamin D levels are lower in individuals with CPP compared to subjects with normal onset of puberty, posing a risk factor for precocious puberty (17, 18, 54, 59–64). Moreover, the proportion of vitamin D deficiency in the CPP population was also higher than in the control group, as shown in **Supplementary Table 1**. Meanwhile, a case-control study identified a correlation between vitamin D levels and uterine volume specifically among girls with ICPP, a relationship not observed in their peers. Girls with ICPP exhibited lower vitamin D levels alongside larger uterine volumes (60). The latest meta-analysis corroborates the notion that severe vitamin D deficiency may elevate the risk of precocious puberty, with patients diagnosed with CPP exhibiting lower levels compared to other types of precocious puberty (65, 66). However, certain clinical studies have failed to establish a significant relationship between vitamin D status and CPP (67, 68). The findings from a cross-sectional study revealed that the vitamin D status among girls aged six to eight with CPP was comparable to that of preadolescent girls. While girls with CPP exhibited significantly elevated levels of parathyroid hormone (67). In the future, further high-quality clinical studies are warranted to validate the relationship between children’s vitamin D levels and CPP. Specifically, large-scale prospective cohort studies or randomized controlled trials with robust methodologies are essential to ensure the reliability of the findings.

## The role of gut microbiota in CPP and vitamin D deficiency

The development of children’s gut microbiota undergoes a dynamic process influenced by various factors. Among these, the mode of delivery, breastfeeding, early exposure to antibiotics, and host-related factors exert significant influence on early life (69–71). While traditionally believed to stabilize within the first three years, recent evidence suggests gut microbiota development continues into childhood and early puberty (22, 72, 73). Puberty, influenced by early nutrition and breastfeeding, sees dynamic changes in gut microbiota, with breast milk offering a protective effect against early puberty, whereas formula feeding is linked to premature puberty (74). Additionally, gender differences in gut microbiota emerge during adolescence and persist into adulthood.

Current research indicates no significant difference in gut microbiota diversity between normal adolescents and prepubertal children, but variations in composition and metabolites exist. A Finnish population-based cohort study found that gut microbiota development during puberty is sex-specific and associated with the timing of puberty in girls. During puberty, the relative abundance of Clostridiales and Bifidobacterium increased, especially Ruminococcaceae, while Bacteroidales decreased (75). A case-control study observed enrichment of Ruminococcus bromii, Ruminococcus gnavus, and Clostridium leptum in ICPP girls, with FSH positively correlated with Fusobacterium and LH with Gemmiger (76). Another cross-sectional study showed higher alpha diversity and upregulated Bacteroides and Faecalibacterium in girls with CPP (77). Li et al. also found that CPP girls had significantly different gut microbiota from normal and overweight girls, with elevated levels of Alistipes, Klebsiella, and Sutterella (78). Functional predictions they conducted suggest peripheral precocious puberty as a transitional stage between ICPP and normal children. Gut bacteria alter sex hormone levels by modifying active-to-inactive ratios, using enzymes like β-glucuronidase (GUS), β-glucosidase, and hydroxysteroid dehydrogenases (HSD) for degradation. Furthermore, The Firmicutes to Bacteroidetes ratio decreases with higher serum hormone levels, indicating an interaction between sex hormones and gut microbes (79). Children with CPP have a higher breast cancer risk in adulthood, and gut microbial β-glucuronidase (gmGUS) inhibitors are a new approach to managing estrogen-related diseases like breast cancer (80, 81).

**Figure 2:**
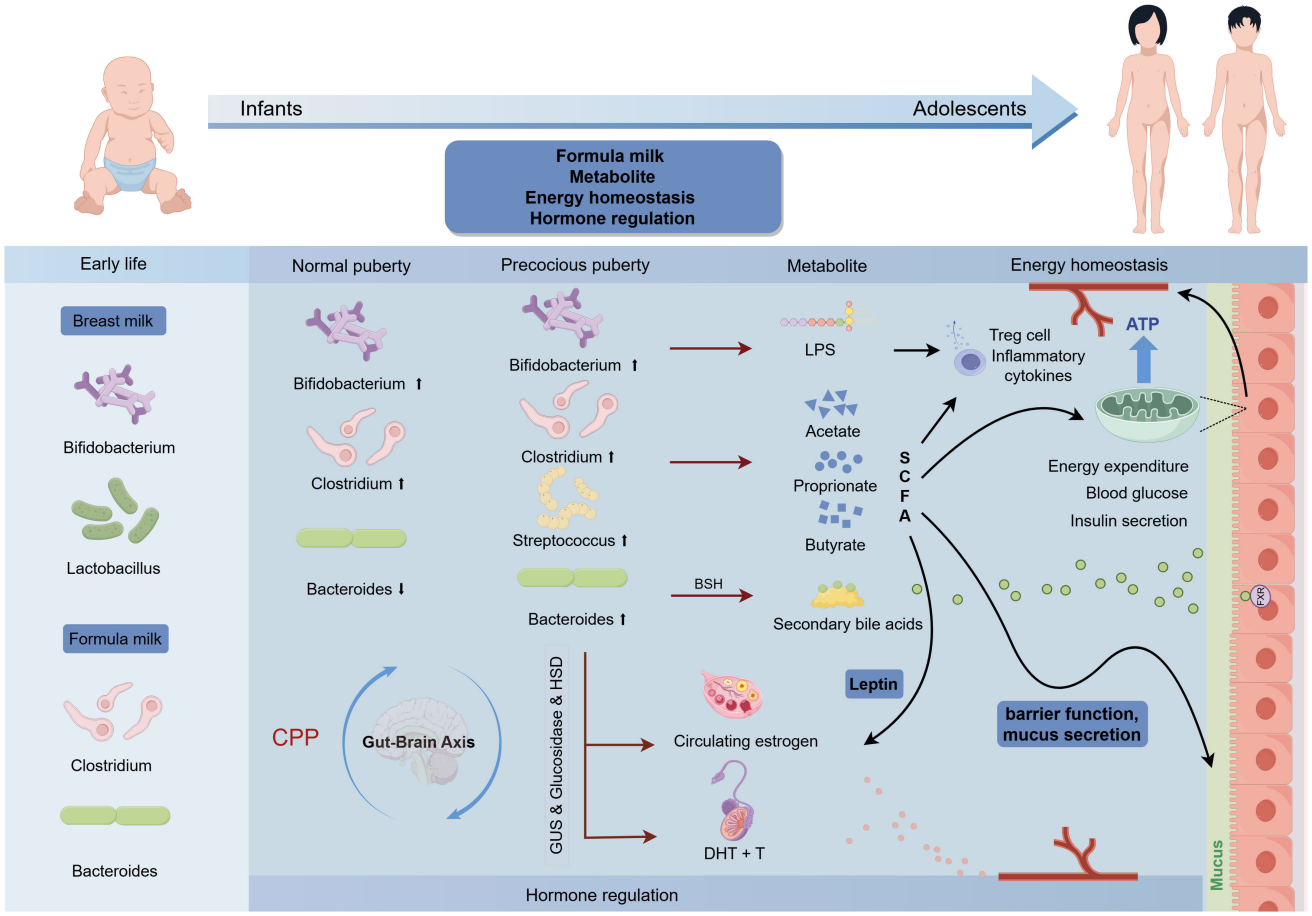
Association of enterotypes in gut microbiota with puberty timing and potential mechanism for CPP. The up and down arrows indicate increase and decrease, respectively.

CPP occurs due to premature activation of the HPGA, and gut microbiota metabolites can influence hypothalamic neurotransmitters and gene expression (82). The gut-brain axis provides a two-way communication pathway via neural, hormonal, and immune pathways, influencing human physiology. Gut microbiota functional analysis suggests the neuroactive compound nitric oxide synthesis is linked to CPP progression (83). Notably, SCFAs, lipopolysaccharides (LPS), and secondary bile acids (BA) are identified as key gut microbial metabolites influencing puberty timing. Dietary carbohydrates fermented by gut microbiota produce SCFAs like acetate, butyrate, and propionate. Bacteroidetes mainly produce acetate and propionate, while Firmicutes produce butyrate (84). *In vitro* studies show butyrate can increase LH and FSH levels, correlating with clinical findings of increased SCFA-producing bacteria in CPP children (85, 86). This increase may promote the expression of leptin and HPG axis-related genes, leading to puberty onset (76, 77, 87). However, excessive SCFAs, particularly butyrate, are associated with promoting intestinal inflammation and insulin resistance, disrupting glucose homeostasis and intestinal mucosal barrier function, which may increase intestinal energy harvesting and promote the development of obesity (88–93). Recent studies indicate that SCFAs modulate GnRH release by the GPR54-PKC-ERK1/2 pathway in the hypothalamus, affecting puberty in female rats on a high-fat diet (HFD). Gut microbiota in precocious puberty rats show dysbiosis and reduced SCFA production. Adding SCFAs to HFD can reverse precocious puberty in rats (94). Butyrate also enhances VDR protein expression while suppressing inflammation (95). The variability between experimental and clinical findings may be due to diet interactions and confounding factors like genetics, environment, and lifestyle. LPS, derived from Gram-negative bacteria, enhances inflammation by inducing macrophage infiltration and proinflammatory cytokines and inhibiting Treg cells. Prevotella triggers TNF-α production, causing secretion of inflammatory cytokines like IL-6, via an LPS-based mechanism (96). The gut microbiome is involved in BA metabolism, regulating secondary BA metabolism, and inhibiting BA synthesis in the liver via farnesoid X receptor (FXR) signaling (97). In addition, BA functions as a signaling molecule, binding to cell receptors. FXR impairs glucose homeostasis (98). The bacterial enzyme bile salt hydrolase (BSH) can modulate FXR signaling by cleaving its antagonist tauro-β-naphthocholic acid (99). Further studies using metagenomics and metabolomics are needed to explore the associations between microbial-derived metabolites (LPS, SCFAs, BAs) and CPP. The mechanisms involving gut microbiota in CPP include differences in early feeding, LPS, SCFAs, energy homeostasis, intestinal barrier maintenance, hormone regulation, and the gut-brain axis, as shown in **Figure 2**.

Vitamin D plays a crucial role in regulating the gut microbiome, and VDR is present in intestinal epithelial cells, immune cells, and gut microbiota. Vitamin D modulates gut microbiome composition and diversity by regulating antimicrobial peptides and immune responses (100). Vitamin D deficiency correlates with gut microbiota imbalance, compromised intestinal defense mechanisms, and increased intestinal permeability (101–103). A genome-wide association study involving 2029 individuals identified two VDR polymorphisms as significant contributors to gut microbiota alterations (104). Vitamin D has shown protective effects on gut microbiota in animal models of inflammatory bowel disease. It induces macrophages to produce antimicrobial peptides, enhances epithelial barrier integrity, regulates the expression of various connexins, and defensins, modulates inflammatory responses, and influences gut microbiota composition (105). Consistent evidence from mouse and human studies indicates an association between vitamin D and gut microbiota beta diversity, but not alpha diversity (106). Clinical studies show that high-dose vitamin D supplementation in adolescent girls improves vitamin D status, increases Firmicutes and Bifidobacterium levels, and decreases Bacteroidetes abundance (27). Vitamin D-deficient children exhibit higher levels of Bacteroides massillensis and Prevotella species (107). Bacteroides and Prevotella influence inflammation by releasing lipopolysaccharides, which activate macrophages. A cross-sectional study found Prevotella more prominent in individuals with high vitamin D intake and lower LPS concentrations in those with better vitamin D status (108). Chronic inflammation from vitamin D deficiency may be linked to CPP through gut microbiota changes. Obesity also impacts gut microbiota. High BMI in children is associated with decreased Bacteroidetes and increased Firmicutes (109). Obese adolescents have a gut microbiota predominantly composed of Firmicutes (94.6%), while Bacteroidetes account for 3.2% (110). However, a cohort study of 295 Dutch children found no correlation between the Firmicutes to Bacteroidetes ratio and BMI (111). Another study on HFD effects on gut microbiota and sexual development in mice found inconsistencies between obesity and precocious puberty-related microbiota changes, highlighting the need to control for obesity when analyzing the gut microbiota-CPP relationship (112).

**Figure 3:**
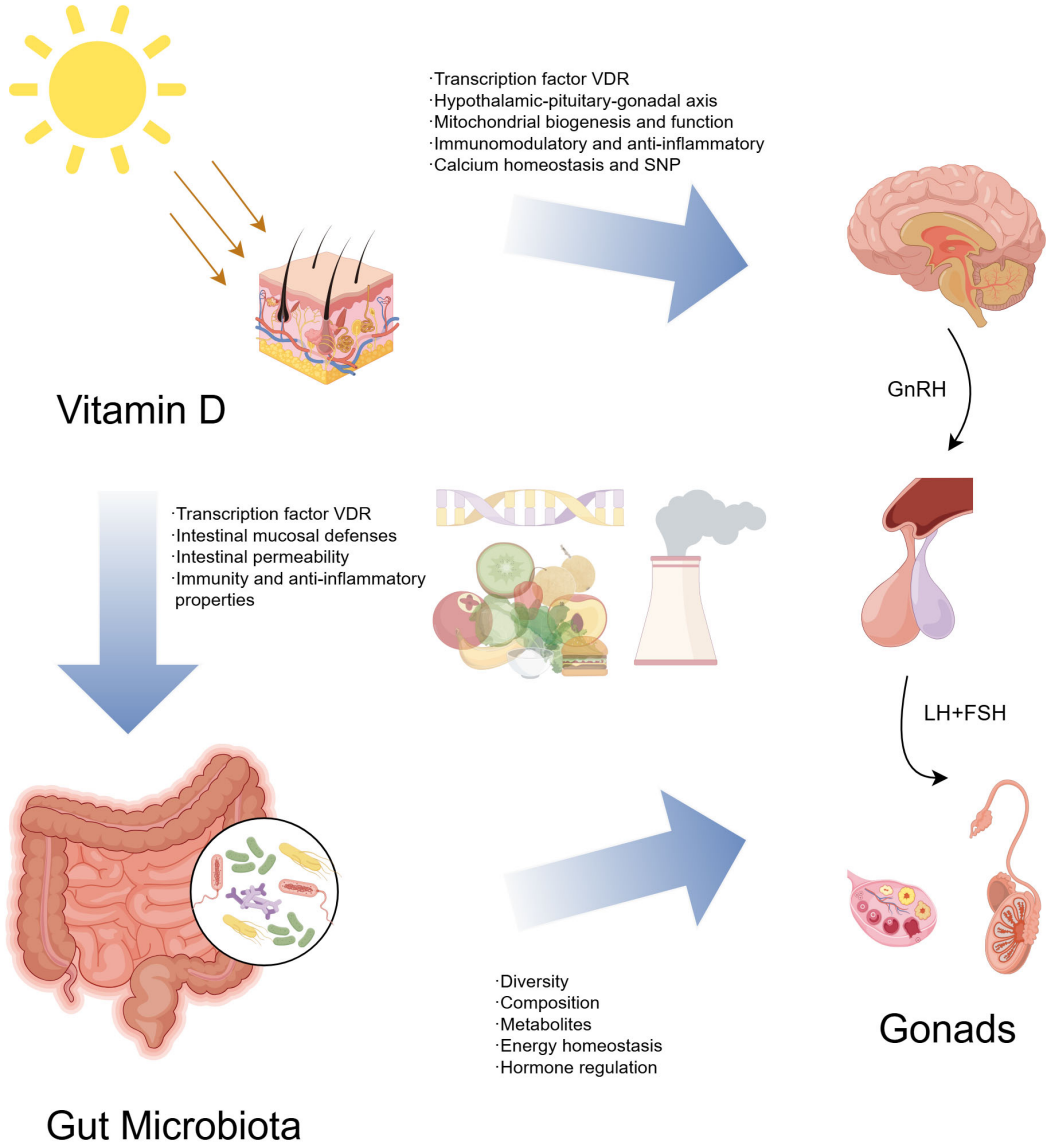
Vitamin D, gut microbiota, and central precocious puberty - a possible three-way axis.

In conclusion, the complex relationship between gut microbiota, vitamin D, and precocious puberty requires further investigation. Key mechanisms likely involve sex hormones, inflammation, immune regulation, and intestinal barrier maintenance. Understanding these interactions is crucial for elucidating CPP pathogenesis and identifying potential treatments. The relationship between vitamin D, gut microbiota, and CPP investigated in this review is shown in **Figure 3**.

## Therapeutic potential of vitamin D supplementation and regulation of gut microbiota on precocious puberty

Recent studies highlight the crucial interaction between vitamin D and gut microbiota in human health, particularly regarding metabolism and immune function. PP stands as a significant health concern in children, prompting increased scrutiny regarding the relationship between CPP and vitamin D deficiency and gut microbiota imbalance.

Previous studies have shown that vitamin D improves insulin sensitivity in adolescents with obesity (113). A recent meta-analysis suggests that combining vitamin D with drug therapy and a short-term/high-dose supplementation strategy is beneficial for sexual hormone and body indices in patients with precocious puberty (65). This observation might explain the persistently high prevalence of vitamin D deficiency despite recommended daily intakes (114, 115). Long-term, low-dose vitamin D does not significantly increase 25(OH)D levels in deficient patients, while high doses rapidly normalize these levels without adverse effects. As shown in **Supplementary Table 2**, clinical trials support the need for high-dose vitamin D supplementation in children with vitamin D deficiency to improve levels and address associated risks (116–130).

Despite these findings, clear and systematic vitamin D supplementation guidelines for clinical practice are lacking. More research is needed to establish guidelines for different pediatric populations. A recent study suggests that lifestyle interventions, such as the Mediterranean diet, may enhance vitamin D levels more effectively than supplementation alone (131). Current research suggests that higher doses of 1,25(OH)2D3 are needed to treat vitamin D deficiency in obese individuals (115). This may be because obesity can reduce circulating 25(OH)D by trapping this lipophilic vitamin in adipose tissue. Addressing weight management or providing vitamin D recommendations based on BMI may be necessary.

Animal studies indicate that insulin resistance linked to gut microbiota induced by HFD can promote early puberty, while microbial remodeling can prevent it (132). Clinical studies have identified Streptococcus as a potential marker for CPP therapy (83). Probiotics have shown promise in upregulating VDR expression, suggesting a synergistic effect with vitamin D. Targeted delivery of vitamins to the colon to modulate gut microbiota is also being explored (133). According to clinical studies, high-protein, complex carb diets may help protect against CPP in girls (77). Probiotic and vitamin D supplementation holds considerable promise for treating precocious puberty, but larger studies are needed to determine optimal dosages and effects. While dietary supplements, including bioactive molecules, show potential, they should never replace dietary or lifestyle modifications. Studies have shown that in addition to vitamin D, other substances like the natural sweetener glycyrrhizin may also have therapeutic potential in CPP, supporting a more comprehensive approach to management in the future (134).

## Conclusion and prospect

This review discussed the extensive evidence demonstrating the importance of the relationship between vitamin D and the gut microbiota in CPP. Vitamin D and the gut microbiota profoundly influence the onset and progression of puberty in many different ways. For example, alterations in vitamin D/VDR signaling have been associated with microbiome dysbiosis, which in turn has been associated with CPP. On the other hand, vitamin D supplementation can also improve microbiome composition in cases of deficiency. While promising, further research is needed to fully understand the potential role of vitamin D and probiotics in modulating the risk of CPP. Correcting vitamin D deficiency and microbiota dysbiosis may offer complementary approaches to standard CPP treatment in the future.

Key questions remain: (1) What novel signal transduction pathways does vitamin D use to regulate gut microbiota and affect puberty, and what are the molecular mechanisms? (2) What are the beneficial impacts of vitamin D on CPP? (3) Why does vitamin D have varying effects on puberty timing in clinical trials versus animal studies? (4) What are the optimal dosage and concentration of vitamin D for CPP patients, and how do they compare with other treatments? More extensive clinical trials are needed to explore these questions and understand the interplay between vitamin D, gut microbiota, and CPP. Ensuring adequate vitamin D levels in CPP children, through diet or supplementation, may be key to maintaining healthy gut microbiota and supporting natural puberty onset.
