# Supplementary material for: Interaction between Vitamin D homeostasis, gut microbiota, and central precocious puberty
Source: Front Endocrinol (Lausanne). 2024 Dec 9;15:1449033. doi: 10.3389/fendo.2024.1449033 (PMC11663660; doi:10.3389/fendo.2024.1449033)
Supplement: Supplementary file 1 [file Table1.docx]

Supplementary Material

# Supplementary Data

Not applicable.

# Supplementary Tables

Table 1. Clinical trials: Differences in Vitamin D Levels Observed in CPP and control group

| Country | Research catalog | Year | Sample size | Age | 25(OH)D_3_ Concentration | Detection method | Proportion of deficiency | Conclusion | Reference |
| --- | --- | --- | --- | --- | --- | --- | --- | --- | --- |
| China | Cross-sectional study | 2023 | SP-CPP  (n=114)  *vs.* RP-CPP  (n=226) | 8.42 (7.90–8.92) *vs.* 8.50 (8.17–8.92) | 16.10 ± 6.84 *vs.* 15.78 ± 6.91 | ECL | 178/340 (overall) | The vitamin D levels were generally deficient or insufficient in girls with CPP but were not related to the  different types of CPP. | [17] |
| China | Cross-sectional study | 2017 | ICPP  (n=280)  *vs.* HC  (n=188) | 8.50 ± 0.87 *vs.* 8.43 ± 0.82 | 19.36 ± 6.15 *vs.* 20.98 ± 7.60 | ECL | 261/280 *vs.* 164/188 | Vitamin D status may be associated with ICPP risk and may have a threshold effect on  ICPP. | [18] |
| China | Cross-sectional study | 2023 | ICPP  (n=221)  *vs.* HC  (n=144) | 8.23 ± 0.81 (overall) | 18.56 ± 6.66 *vs.* 25.15 ± 7.84 | ECL | 149/221 *vs .*31/114 | Vitamin D deficiency is an independent risk factor for ICPP in girls. | [54] |
| Korea | Cross-sectional study | 2014 | CPP  (n=60)  *vs.* HC  (n=30) | 8.3 ± 0.53 *vs.* 7.6 ± 1.3 | 17.1 ± 4.5 *vs.* 21.2 ± 5.0 | RIA | 42/60 *vs.* 13/30 | Vitamin D levels may be associated with precocious puberty. | [59] |
| China | Cross-sectional study | 2023 | ICPP  (n=278)  *vs.* HC  (n=239) | 7.71 (7.00–8.35) *vs.* 7.58 (7.00–8.00) | 31.98 (23.28–44.73) *vs.* 34.88 (25.47–45.90) | ECLIA | 126/278 *vs.* 85/239 | ICPP girls often exhibit lower vitamin D levels and increased  uterine volume. | [60] |
| China | cohort study | 2022 | CPP  (n=58)  *vs.* HC  (n=124) | 7.31 ± 1.00 *vs.* 7.21 ± 0.91 | 13.76 ± 4.10 *vs.* 27.61 ± 8.64 | ECL | - | The increased incidence of PP in girls during  the COVID-19 pandemic may be related to Vitamin D deficiency. | [61] |
| Iran | Cross-sectional study | 2018 | CPP  (n=28)  *vs.* HC  (n=30) | 7.1 ± 1.3 *vs.* 6.9 ± 1.2 | 12.9 ± 7.8 *vs.* 15.2 ± 5.9 | ELISA | 9/28 *vs.* 5/30 | Vitamin D  deficiency was more common in girls with central  precocious puberty than those with normal sexual  maturation. | [62] |
| China | Cross-sectional study | 2022 | ICPP  (n=93)  *vs.* HC  (n=93) | 7.0 (7.0–8.0) *vs.* 7.0 (7.0–8.0) | 20.55 (17.53–23.9) *vs.* 26.10 (22.43–30.48) | ECL | - | Significant differences in  serum P1NP, β-CTX, Vit D, and E2 between ICPP girls and healthy girls. | [63] |
| China | cohort study | 2024 | ICPP  (n=12)  *vs.* PT  (n=38) | 6.75±1.06 *vs.* 6.66±0.78 | 79.22 ± 6.98 *vs.* 83.85 ± 7.56 | high-performance liquid chromatography | - | Vitamin D level began to decrease half a year after the identification of breast development in children from the conversion group, which was significantly lower than the normal level. | [64] |
| Spain | Cross-sectional study | 2022 | CPP  (n=78)  *vs.* HC  (n=137) | 7.4 ± 0.5 *vs.* 7.5 ± 0.7 | 25.4 ± 8.6 *vs.* 28.2 ± 7.4 | ECLIA | 12/78 *vs.* 16/137 | Vitamin D status in 6- to 8-year-old girls with CPP is similar to that in prepubertal girls. | [67] |
| Italy | cohort study | 1995 | CPP  (n=12)  *vs.* HC  (n=12) | 5.9 ± 2.1* | 23.11 ± 4.87 *vs.* 23.5 ± 3.72 | competitive protein-binding assay | - | The parameters  studied remained normal in patients who received  the GnRH analog D-Trp. | [68] |

SP-CPP, rapidly progressive central precocious puberty; RP-CPP, slowly progressive central precocious puberty; ICPP, idiopathic central precocious puberty; HC, healthy control; ECL, electrochemiluminescence; RIA, radioimmunoassay; ECLIA, electrochemiluminescence immunoassay; ELISA, enzyme-linked immunosorbent assay; *The healthy control group consisted of age-matched children of the same gender. PT, simple premature thelarche.

Table 2. Clinical trials: vitamin D supplementation strategies in various children groups

| Country | Research catalog | Year | Population | Age | Vitamin D^3^ Dosage | Duration of Treatment | Outcome | Reference |
| --- | --- | --- | --- | --- | --- | --- | --- | --- |
| America | RCT | 2013 | Obese adolescents (n=35) | 14.1 ± 2.8 | 4000 IU/day | 6 months | Participants supplemented with vitamin D3 had increases in serum 25(OH)D concentrations. | [116] |
| America | RCT | 2022 | Racially diverse school children (n=604) | 8-15 | 600 IU/day  1000 IU/day  2000 IU/day | 6 months | The most beneficial effects are observed with supplementation of 600 IU/d, which aligns most closely with the current daily vitamin D intake recommendations for children. | [117] |
| Brazil | RCT | 2021 | Hypertriacylglycerolemia children (n=44) | 4-11 | 1000 IU/day | 12 weeks | Vitamin D supplementation improves children's lipid profile without changing body composition.  Vitamin D supplementation was beneficial to children even with sufficient 25-hydroxyvitamin D. | [118] |
| Saudi Arabia | RCT | 2019 | Healthy adolescents with 25(OH)D levels <50 nmol/l* (n=535) | 12-18 | 1000 IU/day  80 IU/day | 6 months | Oral vitamin D supplementation is clinically superior to fortified foods in reducing MetS risk factors and can reduce the incidence of MetS in Arab adolescents. | [119] |
| America | RCT | 2015 | Overweight and obese adolescents* (n=31) | 11-17.99 | 150000 IU/12 weeks | 24 weeks | The dose of 150,000 IU given every 12 weeks failed to significantly increase serum 25OHD levels and did not alter serum inflammatory or metabolic markers. | [120] |
| Denmark | RCT | 2018 | Healthy children (n=119) | 4-8 | 10 ug/day^#^  20 ug/day^#^ | 20 weeks | Preventing the winter decline in serum 25(OH)D with daily vitamin D3 supplementation of 10 or 20 µg had no cardiometabolic effects in healthy 4- to 8-y-old Danish children. | [121] |
| Denmark | RCT | 2018 | Healthy children (n=110) | 14-18 | 10 ug/day^#^  20 ug/day^#^ | 20 weeks | vitamin D3 supplementation at the currently recommended dietary intakes increased winter-time serum 25(OH)D concentrations but did not affect markers of cardiometabolic risk in healthy 14- to 18-y-old white adolescents. | [122] |
| America | RCT | 2013 | Healthy adolescents (n=53) | 11-19 | 200 IU/day  1000 IU/day | 11 weeks | In healthy adolescents with baseline vitamin D sufficiency, supplementation with vitamin D3 doses of 200 and 1,000 IU for 11 weeks did not increase serum 25(OH)D levels, with no significant difference observed between treatment arms. | [123] |
| America | RCT | 2014 | Obese adolescents* (n=44) | 12-18 | 2000 IU/day | 12 weeks | 12 weeks of vitamin D3 supplementation in obese adolescents with 2,000 IU once daily resulted in a modest increase in 25(OH)D concentration in obese adolescents but did not affect the lipid profile and markers of insulin resistance and inflammation. | [124] |
| America | RCT | 2015 | Obese adolescents (n=47) | 12-18 | 400 IU/day  2000 IU/day | 12 weeks | No effect from vitamin D3 supplementation β-cell function or insulin action in obese nondiabetic adolescents with relatively good vitamin D status. | [125] |
| Iran | RCT | 2014 | obese children and adolescents (n=43) | 10-16 | 30000 IU/week | 12 weeks | Vitamin D supplementation has favorable effects on reducing insulin resistance and cardiometabolic risk factors in obese children. | [126] |
| Iran | RCT | 2020 | high school male students* (n=71) | 17 | 50000 IU/month | 6 months | A supplementation regimen of (50,000 IU/month) vitamin D in a context with high rates of vitamin deficiency has shown positive impacts on the serum vitamin D, lipid profile and inflammatory biomarkers in healthy adolescent boys. | [127] |
| Argentina | RCT | 2013 | Indian children (n=90) | 10.21 ± 2.07 | 5000 IU/week | 8 weeks | The supplement of 5,000 U/week of vitamin D did not result in serum 25(OH)D sufficient concentrations (>30 ng/ml) in any of the treated children. However, raising 25(OH)D levels from deficient to insufficient in this cohort improved the HDL-C level. | [128] |
| Poland | RCT | 2020 | Overweight or obese vitamin D deficient children* (n=109) | 6-14 | 1200 IU/day | 26 weeks | Vitamin D supplementation does not affect body weight reduction in children and adolescents with vitamin D insufficiency undergoing a weight management program. | [129] |
| Mongolia | RCT | 2023 | School-aged children* (n=8851) | 6-13 | 14 000 IU/week | 3 years | Oral vitamin D3 supplementation at a dose of 14 000 IU per week for 3 years was effective in elevating 25(OH)D concentrations but did not influence growth, body composition, or pubertal development. | [130] |

RCT, Randomized controlled trial; ^#^1ug = 40IU; * Trials were conducted in participants with vitamin D deficiency
